# High-power short-duration vs. conventional catheter ablation for atrial fibrillation: a meta-analysis and trial sequential analysis of randomized controlled trials

**DOI:** 10.3389/fcvm.2025.1703573

**Published:** 2025-12-15

**Authors:** Lin He, Yi He, Shuang-Yan Zhang, Ji-Lin Hu, Hai-Ping Cao, Wei Wang, Jing-Hong Zhao, Peng-Yu Zhong

**Affiliations:** Department of Cardiology, Beijing Anzhen Nanchong Hospital of Capital Medical University & Nanchong Central Hospital (The Second Clinical College of North Sichuan Medical College), Nanchong, Sichuan, China

**Keywords:** high-power short-duration ablation, low-power long-duration ablation, atrial fibrillation, randomized controlled trials, meta-analysis

## Abstract

**Background:**

High-power short-duration (HPSD) ablation has emerged as an alternative to traditional low-power long-duration (LPLD) ablation. However, the safety and efficacy of HPSD remain controversial. This study aimed to evaluate the effectiveness and safety of HPSD in the ablation treatment of atrial fibrillation (AF).

**Methods:**

Randomized controlled trials (RCTs) comparing high-power short-duration (HPSD) and low-power long-duration (LPLD) ablation were retrieved from PubMed, Web of Science, EMBASE, and the Cochrane Library up to 20 May 2025. Statistical analysis was performed using RevMan 5.4 software. The risk ratio (RR) was used as the effect size for dichotomous variables, and the mean and standard deviation were used as the effect sizes for continuous variables.

**Results:**

A total of eight RCTs involving 1,024 patients were included. HPSD was significantly associated with a reduction in total procedure time [mean differences (MD), −20.33; 95% CI: −30.46 to −10.21; *P* < 0.0001], pulmonary vein isolation time (MD, −22.01; 95% CI: −27.10 to −16.95; *P* < 0.00001), radiofrequency time (MD, −10.38; 95% CI: −12.47 to −8.29; *P* < 0.00001), and AF recurrence (RR, 0.51; 95% CI: 0.36–0.74; *P* = 0.0004, *I*^2^ = 14%, *P* = 0.32). However, no significant differences were observed between HPSD and LPLD ablation in all atrial arrhythmias recurrence (RR, 1.06; 95% CI: 0.75–1.49; *P* = 0.74, *I*^2^ = 40%, *P* = 0.15), the incidence of esophageal lesions (RR, 1.21; 95% CI: 0.55–2.64; *P* = 0.63), any complications (RR, 1.37; 95% CI: 0.76–2.45; *P* = 0.29), first-pass left pulmonary vein (LPV) isolation (RR, 0.96; 95% CI: 0.91–1.01; *P* = 0.10, *I*^2^ = 47%, *P* = 0.11), and first-pass right pulmonary vein (RPV) isolation (RR, 1.01; 95% CI: 0.69–1.48; *P* = 0.97, *I*^2^ = 75%, *P* = 0.003).

**Conclusion:**

Compared with LPLD ablation, HPSD ablation was significantly associated with a reduction in total procedure time, PVI time, and radiofrequency time. HPSD ablation did not increase the risk of complications compared with LPLD ablation, but it may be underpowered to detect rare adverse events, necessitating additional large-scale RCTs to validate the safety profile of HPSD.

**Systematic Review Registration:**

https://www.crd.york.ac.uk/PROSPERO/view/CRD42023471797, PROSPERO CRD42023471797.

## Introduction

Atrial fibrillation (AF), the most common type of arrhythmia, is often not directly fatal but can lead to the development of heart failure and cerebral infarction, severely threatening patients' long-term prognosis. Current guidelines recommend catheter ablation for symptomatic AF patients who are refractory or intolerant to antiarrhythmic drugs. Additionally, for patients with paroxysmal AF, catheter ablation can serve as an initial rhythm-control strategy that aims to alleviate symptoms and reduce the risk of progression to persistent AF (1). Studies have shown that catheter ablation is more effective than antiarrhythmic drugs in maintaining normal sinus rhythm (2, 3).

Existing research suggests that the primary triggers for AF initiation and perpetuation are the ectopic electrical activities originating from the pulmonary veins of the left atrium (4). Therefore, the goal of AF ablation is to isolate the pulmonary veins by creating circumferential ablation lesions around the ostia of the pulmonary veins. Radiofrequency ablation cauterizes cardiac tissue by delivering thermal energy, and its efficacy and safety depend on the formation of full-thickness and durable lesions. However, excessive ablation can cause complications such as esophageal thermal injury and cardiac perforation. Key factors influencing the size and depth of lesions include radiofrequency ablation power, duration, catheter stability, and contact force. Conventional low-power long-duration (LPLD) ablation (20–35 W, 20–40 s per lesion) is often limited by inadequate tissue-electrode contact, often leading to non-transmural lesion formation. In contrast, emerging high-power short-duration (HPSD) protocols (40–90 W, ≤15 s per lesion) leverage the biophysics of dominant resistive heating to generate broader superficial lesions while maintaining transmural efficacy (4). Multiple randomized controlled trials (RCTs) have compared the outcomes of HPSD and LPLD (5–8), but these studies have reported divergent conclusions.

This meta-analysis aims to systematically evaluate the efficacy and safety of HPSD in AF radiofrequency ablation, synthesizing existing evidence to clarify its clinical utility and unresolved controversies.

## Method

This study was conducted in strict accordance with the PROSPERO protocol (registration number: CRD42023471797). Throughout the research process, it fully complied with the guidelines of the PRISMA statement and the relevant specifications outlined in the Cochrane Handbook for Systematic Reviews and Meta-Analyses, thereby ensuring scientific validity, methodological rigor, and transparency (9).

### Data sources and search strategy

Two researchers (LH and J-LH) independently performed systematic searches across multiple databases, including PubMed, EMBASE, Web of Science, and the Cochrane Library. The search scope covered the entire timeline from each database's inception to 20 May 2025. For each database, a tailored search strategy was designed, incorporating a combination of Medical Subject Headings (MeSH) and free-text terms—such as “high-power short-duration atrial fibrillation” and “low-power long-duration atrial fibrillation.” The full details of each search strategy are documented in the Supplementary Material for reference.

### Inclusion and exclusion criteria

This study employed rigorous PICO (Population, Intervention, Comparison, Outcomes) criteria for literature screening. The inclusion criteria were specified as follows:

1. Population: adult patients with paroxysmal or persistent AF.

2. Intervention: high-power short-duration ablation.

3. Comparison: low-power long-duration ablation.

4. Outcomes: Studies must report at least one primary outcome [total procedure time, pulmonary vein isolation (PVI) time, radiofrequency application time] or secondary outcomes (atrial fibrillation recurrence, atrial arrhythmia recurrence, first-time left/right PVI rate, esophageal lesions, and any complication incidence).

Study types such as letters, essays, editorials, book chapters, cohort studies, case series, case reports, single-arm studies, animal studies, *in vitro* studies, preliminary studies, and studies with data available only in abstracts.

### Study screening and data extraction

Initially, two researchers (LH and P-YZ) independently screened literature titles and abstracts using the Covidence online platform. Duplicate studies were first removed, after which the remaining literature underwent a second independent screening round. For discrepancies in the screening process, two additional researchers (J-HZ) engaged in discussions and made final judgments based on the predefined inclusion and exclusion criteria. Following the determination of included studies, two researchers independently extracted data. The extracted information encompassed design, number of participating centers, total sample size, power settings, key inclusion criteria, and follow-up duration. Patient baseline characteristics: age, CHADS₂-VASc scores, left ventricular ejection fraction, and Atrial fibrillation type (paroxysmal/persistent).

### Risk of bias assessment and certainty of evidence evaluation

Two researchers independently assessed the quality of included studies using the Cochrane Risk of Bias (RoB) 2 tool, evaluating potential bias risks across multiple domains: randomization process, deviation from intended interventions, missing outcome data, outcome measurement, and selective reporting of results (10, 11). Discrepancies in evaluations were resolved through discussion. Concurrently, a third researcher appraised the certainty of evidence for each outcome indicator following GRADE guidelines, documenting evaluation rationale and decision-making processes in detail to ensure accurate assessment of research evidence quality (12).

### Statistical analysis

RevMan v5.4 software was employed for statistical analyses. For dichotomous variables, risk ratios (RR) with 95% confidence intervals (CIs) were used for combined analysis, while continuous variables were summarized using mean differences (MD) and their 95% CIs. Heterogeneity across studies was evaluated via the chi-square test, with the *P*-value assessing the presence of heterogeneity and *I*^2^ quantifying its degree. *P* < 0.1 indicated intragroup heterogeneity, and *P* ≤ 0.05 denoted intergroup heterogeneity. *I*^2^ values were interpreted as follows: <25% (mild), 25%–50% (moderate), and >50% (severe) heterogeneity (13). When *P* < 0.05, severe significant heterogeneity (*I*^2^ > 50%) was considered to exist, random-effects model was used.

In cases of significant intergroup heterogeneity, sensitivity analyses were conducted by sequentially excluding each included study to identify the source of heterogeneity and assess its impact on result stability. Additionally, subgroup analyses were performed based on the power settings in the HPSD group (≥50 W vs. <50 W) to further explore differences in study outcomes under varying power conditions. To explore potential sources of heterogeneity, meta-regression was performed using the meta package (version 6.5-1) in R software (version 4.4.1). Moreover, publication bias was initially assessed via visual inspection of funnel plots and Egger's test.

To mitigate the risk of Type I errors stemming from repeated significance testing or inadequate sample size, we applied trial sequential analysis (TSA) to gauge the stability of the combined effect. The analysis was carried out using TSA software (version 0.9.5.10), adopting a two-tailed testing approach with a Type I error rate (*α*) set at 0.05 and statistical power (1 − *β*) at 80%. The relative risk reduction (RRR) was derived from data of recently published large-scale RCTs, and the required information size (RIS) was computed based on this. Evidence was considered adequate and statistically reliable when the cumulative *Z*-curve either crossed the TSA monitoring boundary or, upon reaching the RIS, exceeded the conventional significance threshold.

## Results

### Search results and baseline characteristics

**Figure 1:**
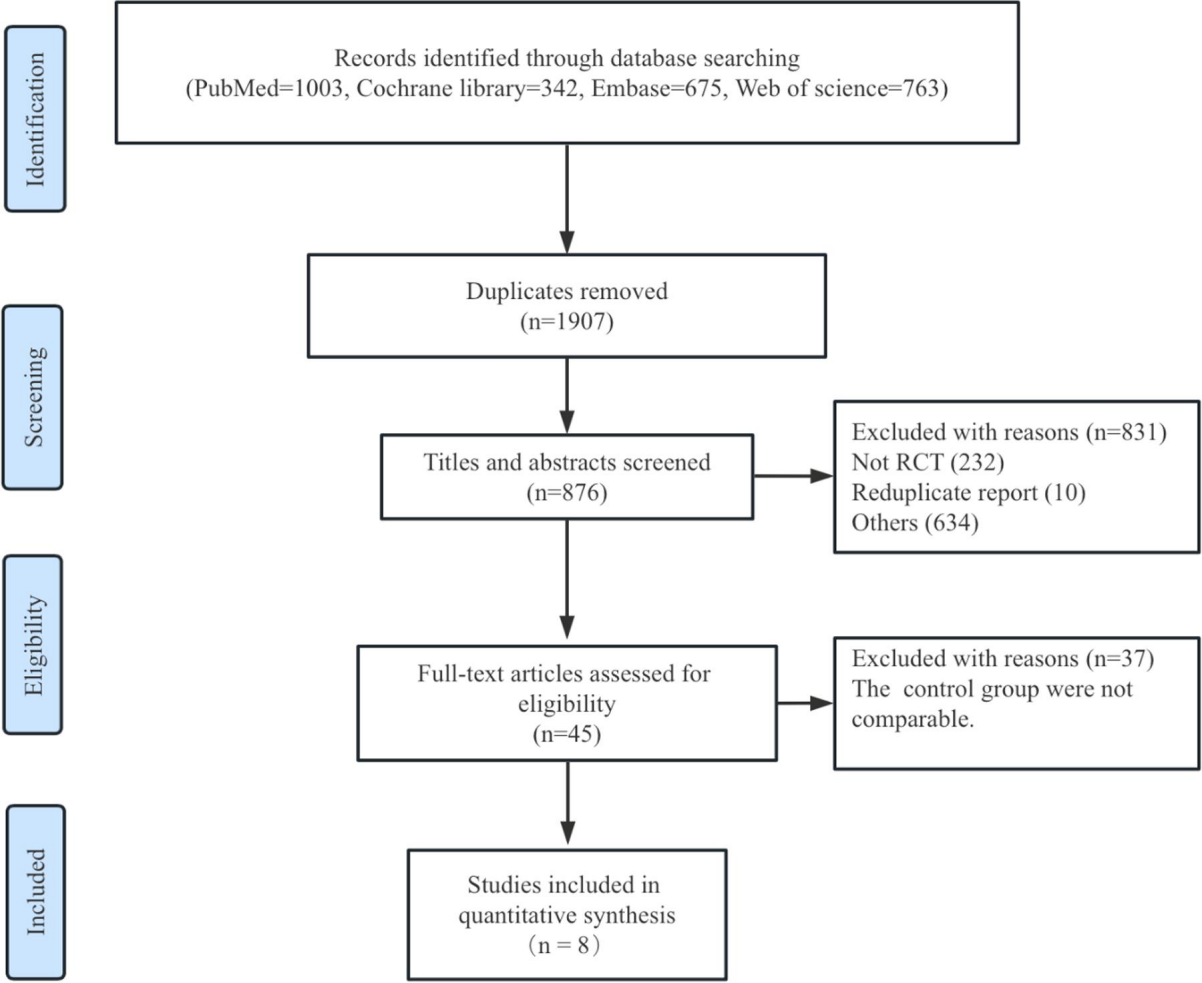
Flow diagram of literature search.

A total of 876 articles were screened, with 45 full texts retrieved and assessed for eligibility. Ultimately, eight randomized trials (14–16) met the inclusion criteria and were incorporated into the final analysis (Figure 1). All included trials enrolled patients with both paroxysmal and persistent AF, except the POWER-AF trial, which focused exclusively on paroxysmal AF patients. The follow-up duration across studies ranged from 6 to 12 months, with the average age of participants in the included trials spanning 59–67 years.

In the LPLD group, radiofrequency ablation energy for the posterior heart wall ranged from 20 to 30 W, except in the POWER PLUS trial, where energy levels were 35–50 W. In the HPSD group, 11 studies used 40–90 W energy across all segments, while the trial by Shin et al. employed 50 W for the anterior wall and 25–30 W for the posterior wall. All trials delivered lesions up to the AI/lesion size index (LSI) target, except for Shin et al.'s study, which used a time-based target. The study by Leo et al. included four groups (two LPLD and two HPSD) with two different LSI targets, necessitating division into subgroups based on LSI target values (four and five, respectively). Baseline characteristics of the study populations are detailed in Table 1.

**Table 1:** The characteristics of included studies and patients.

Trials	Number of patients	Type of AF	LPLD	HPSD	Proportion of paroxysmal AF (%)	Mean age	CHA2DS2-VASc (Mean) LPLD/HPSD	LVEF LPLD/HPSD	Follow-up duration (months)
POWER-AF, 2021	48/48	Paroxysmal AF	25 W posterior/roof, 35 W anterior to AI target	45 W full-wall to AI target	100/100	64/61	1/1	NA	6
Shin et al., 2020	50/50	Paroxysmal/persistent AF	30 W/40 s anterior, 25–30 W/20 s posterior	50 W/10 s anterior, 25–30 W/20 s posterior	50/48	59/59	1.7/1.6	58.9/55.7	12
SHORT-AF, 2023	29/31	Paroxysmal/persistent AF	30 W ridge/septal, 25 W posterior to AI/LSI target	50 W full-wall to AI/LSI target	59/55	67/63	2/2	60/60	12
PILOT-AF, 2020	20/20	Paroxysmal/persistent AF	20 W posterior, 40 W other regions to LSI4 target	40 W full-wall to LSI4 target	40/45	60/60	1/1	60/60	12
	20/20	Paroxysmal/persistent AF	20 W posterior, 40 W other regions to LSI5 target	40 W full-wall to LSI5 target	40/30	61/56	1/2	60/57.9	12
Hi-Lo HEAT, 2023	44/44	Paroxysmal/persistent AF	25 W posterior, 40 W anterior to AI/LSI target	40–50 W full-wall to AI/LSI target	34/48	63/60	1.5/2	54.6/55.4	12
Francke et al., 2021	100/20	Paroxysmal/persistent AF	20 W posterior, 40 W other regions to AI target	50W full-wall to AI target	49/45	66/66	2.8/3.2	54.2/59.5	12
POWER PLUS, 2023	90/90	Paroxysmal/persistent AF	35–50 W to AI target	90 W/4 s temperature-controlled mode	91/83	64/62	1.7/1.5	82/83	6
POWER FAST III, 2025	147/153	Paroxysmal/persistent AF	25–40 W to AI/LSI target	70W/9–10 s to AI/LSI target	65/63	60/61	1/0	60/60	12

AF, atrial fibrillation; AI, ablation index; LSI, lesion size index; LPLD, low-power long-duration; HPSD, high-power short-duration; LVEF, left ventricular ejection fraction.

#### Procedure time

**Figure 2:**
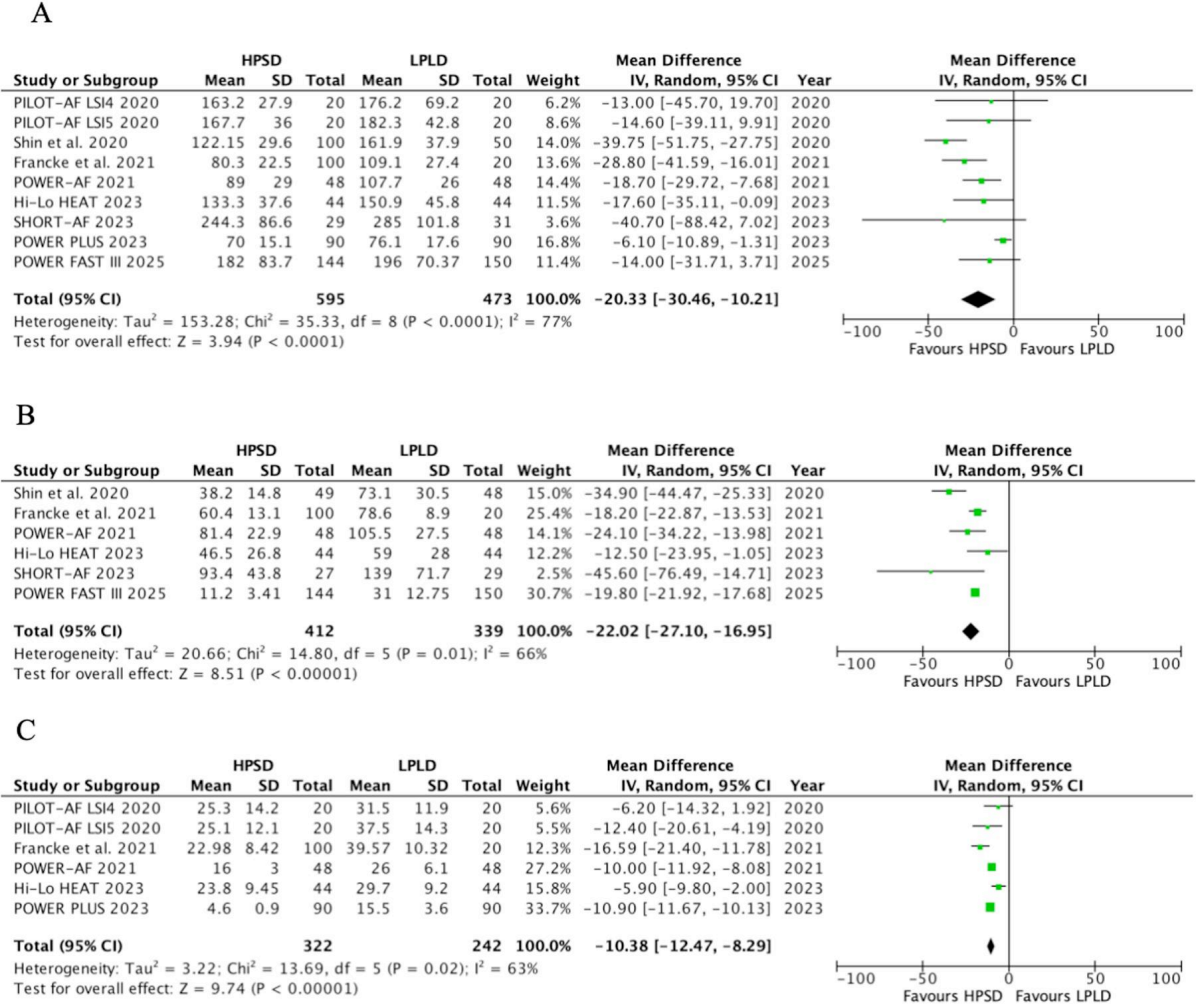
Pooled analyses of HPSD vs. LPLD for the procedure time (based on random-effects models). **(A)** Total procedure time; **(B)** PVI time; **(C)** radiofrequency application time.

The procedure time included the total procedure time, PVI time, and radiofrequency application time, and the results are shown in Figure 2. HPSD demonstrated a significantly shorter total procedure time compared with LPLD (MD, −20.33; 95% CI: −30.46 to −10.21; *P* < 0.0001), with substantial heterogeneity observed (*I*^2^ = 77%, *P*_heterogeneity_ < 0.0001) (Figure 2A). Subgroup analysis by power intensity revealed (Supplementary Figure S1) the following: in the 40–50 W HPSD subgroup, total procedure time was reduced by 17.58 min (MD, −17.58; 95% CI: −26.01 to −9.63; *P* < 0.0001). In the 50–90 W HPSD subgroup, a similar reduction was observed (MD, −23.55; 95% CI: −40.56 to −6.54; *P* = 0.007). No significant heterogeneity was detected between the two subgroups (*P*_interaction_ = 0.54, *I*^2^ = 0%).

HPSD was associated with shorter PVI time (MD, −22.01; 95% CI: −27.10 to −16.95; *P* < 0.00001, *I*^2^ = 66%, *P* = 0.01) (Figure 2B) and radiofrequency application time (MD, −10.38; 95% CI: −12.47 to −8.29; *P* < 0.00001, *I*^2^ = 63%, *P* = 0.02) (Figure 2C).

To explore the sources of heterogeneity, further meta-regression results showed that AF type, HPSD power selection, and CHA2DS2-VASc score might be the main factors contributing to the heterogeneity of total procedure time, PVI time, and radiofrequency application time (*P* < 0.05) (Supplementary Tables S3–S5).

#### AF recurrence

**Figure 3:**
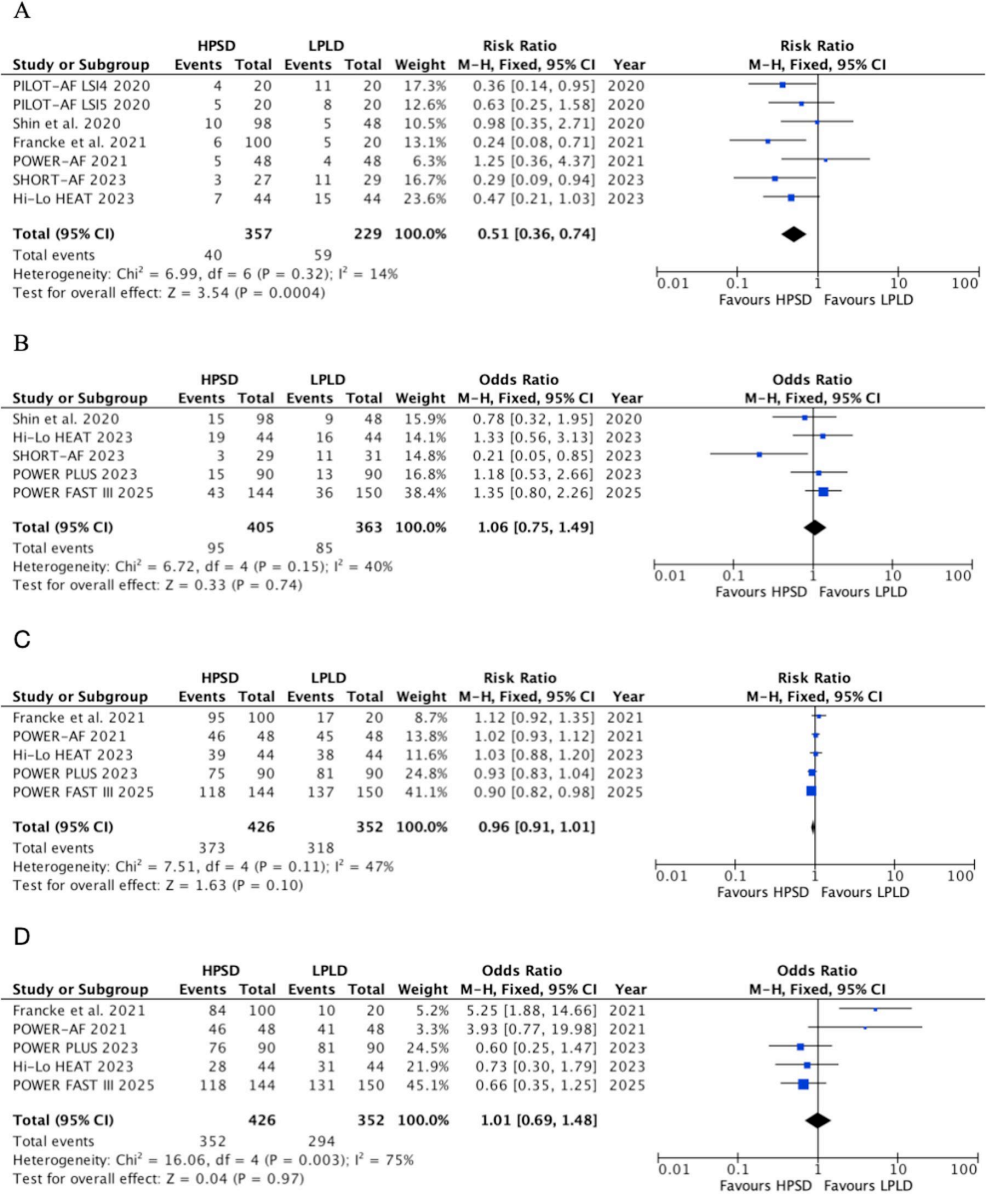
Pooled analyses of HPSD vs. LPLD for the efficacy outcomes (based on fixed-effects models). **(A)** Atrial fibrillation recurrence; **(B)** atrial arrhythmia recurrence; **(C)** first-time left pulmonary vein isolation rate; **(D)** first-time right pulmonary vein isolation rate.

A total of seven trials reported the incidence of AF recurrence (Figure 3A). HPSD significantly reduced the risk of AF recurrence (RR, 0.51; 95% CI: 0.36–0.74; *P* = 0.0004) with low heterogeneity (*I*^2^ = 14%, *P* = 0.32). Subgroup analysis by power intensity revealed (Supplementary Figure S2) the following: in the 50–90 W HPSD subgroup, AF recurrence was reduced by 54% (RR, 0.46; 95% CI: 0.25–0.83; *P* = 0.01), with moderate heterogeneity (*I*^2^ = 51%, *P* = 0.13). In the 40–50 W HPSD subgroup, a 45% reduction was observed (RR, 0.55; 95% CI: 0.35–0.88; *P* = 0.01), with no heterogeneity (*I*^2^ = 0%, *P* = 0.46).

#### All atrial arrhythmias recurrence

Five trials reported the risk of all atrial arrhythmias recurrence (Figure 3B). No significant difference was found in the composite endpoint of all atrial arrhythmias recurrence between groups (RR, 1.06; 95% CI: 0.75–1.49; *P* = 0.74; *I*^2^ = 40%, *P* = 0.15). Sensitivity analysis excluding the SHORT-AF trial revealed no effect of HPSD on arrhythmia recurrence (RR, 1.21; 95% CI: 0.84–1.73; *P* = 0.30; *I*^2^ = 0%, *P* = 0.78) (Supplementary Figure S3).

#### First-pass PVI

Five trials reported the first-pass PVI (Figure 3C,D). HPSD showed a non-significant trend toward lower first-pass LPV isolation rates compared with LPLD (RR, 0.96; 95% CI: 0.91–1.01; *P* = 0.10), with moderate heterogeneity (*I*^2^ = 47%, *P* = 0.11). No significant difference was observed in first-pass RPV isolation rates (RR, 1.01; 95% CI: 0.69–1.48; *P* = 0.97), although high heterogeneity was noted (*I*^2^ = 75%, *P* = 0.003). The further sensitivity analysis was performed by excluding the study of Francke et al. (Supplementary Figure S4). HPSD did not increase the incidence of first-pass RPV isolation (RR, 0.96; 95% CI: 0.90–1.03; *P* = 0.24, *I*^2^ = 50%, *P* = 0.11).

### Safety outcomes

**Figure 4:**
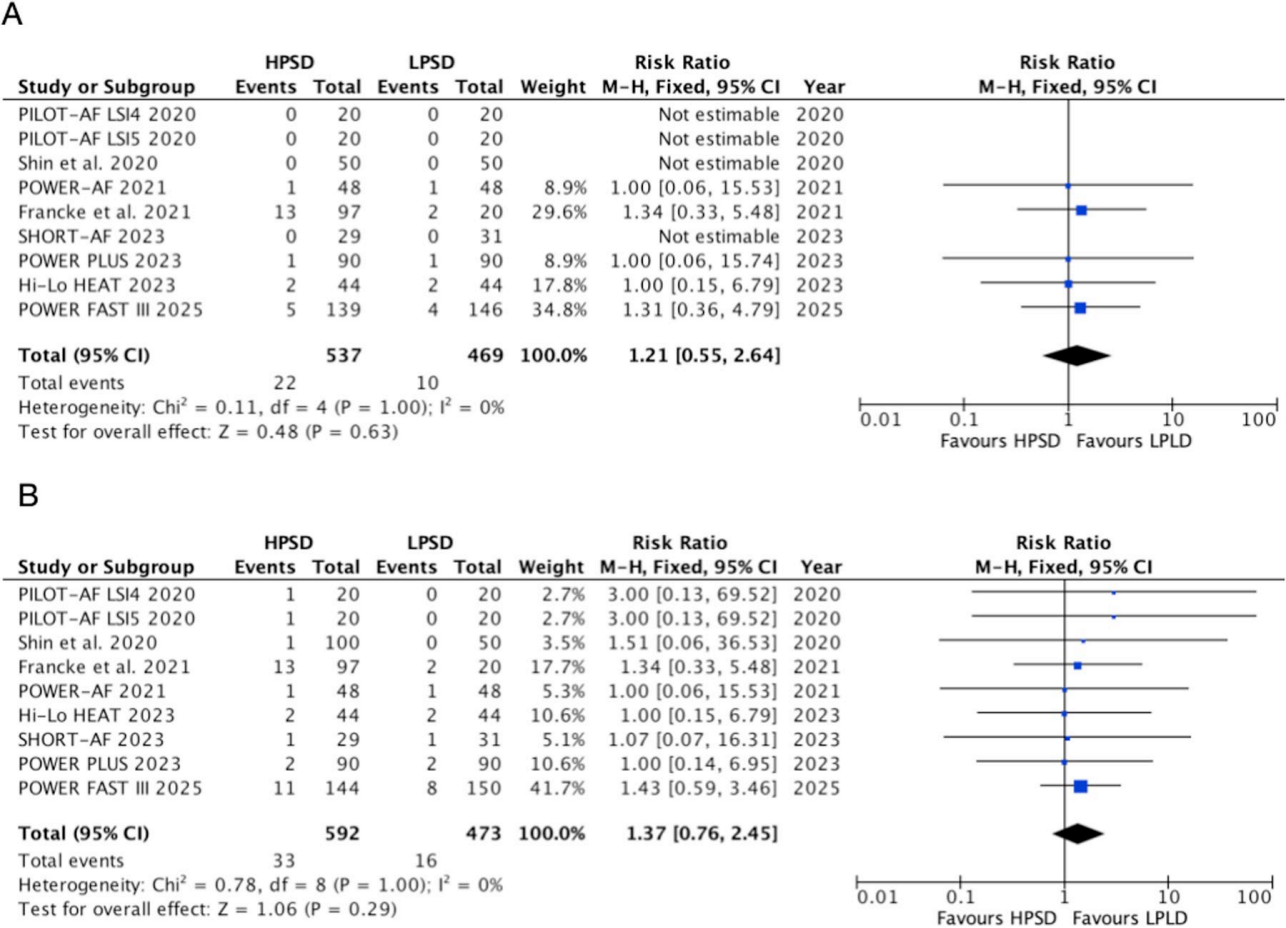
Pooled analyses of HPSD vs. LPLD for the safety outcomes (based on fixed-effects models). **(A)** Esophageal lesions; **(B)** any complication incidence.

HPSD did not significantly increase the incidence of esophageal lesions (RR, 1.21, 95% CI: 0.55–2.64, *P* = 0.63, *I*^2^ = 0%, *P* = 1.00) and any complications (RR, 1.40, 95% CI: 0.75–2.59, *P* = 0.29, *I*^2^ = 0%, *P* = 1) compared with LPLD (Figure 4). In HPSD (50–90 W) subgroups, HPSD not increase the risk of esophageal lesions (RR, 1.29; 95% CI: 0.52–3.16; *P* = 0.58, *I*^2^ = 0%, *P* = 0.98) and any complications (RR, 1.33; 95% CI: 0.69–2.58; *P* = 0.39, *I*^2^ = 0%, *P* = 1.00) (Supplementary Figures S5 and S6). Similarly, no difference was found in the HPSD (40–50 W) subgroups.

#### Risk of bias assessment and certainty of evidence evaluation

The quality assessment of each trial and the quality assessment of GRADE evidence are shown in Supplementary Figure S7 and Supplementary Table S2. There is a low risk of bias in selection, detection, and reporting, but there is a high risk of performance in three out of seven trials. The quality assessments of GRADE evidence of total procedure time is moderate, and other outcomes are high.

#### Trial sequential analysis and publication bias

The trial sequential analysis of each outcome was performed, and the results are shown in Supplementary Figure S8 and Supplementary Table S6. The cumulative *Z*-curves of total procedure time, PVI time, radiofrequency application time, and AF recurrence are beyond the TSA boundary, in which PVI time and radiofrequency application time reach the expected sample size, 199 and 358, respectively. The others did not arrive at the conventional boundary and expected sample size (Supplementary Figure S8 and Supplementary Table S6). The funnel plots are shown in Supplementary Figure S9, and all the points of each outcome are almost symmetrically distributed, which testifies that no publication bias was found. Egger's test, presented in Supplementary Table S7, indicates that the *P*-values were >0.05 for all outcomes, suggesting no evidence of publication bias.

## Discussion

This meta-analysis comprehensively compared the efficacy and safety of HPSD and LPLD ablation for AF. Compared with LPLD, HPSD was associated with a lower rate of AF recurrence and reduced procedure time, PVI time, and radiofrequency application time. Additionally, no statistical differences were observed between the two ablation strategies in first-pass isolation success rate or overall complication incidence.

Radiofrequency ablation has become a mainstream treatment for AF, operating on the principle of generating thermal lesions through the tip of an ablation catheter to block ectopic pacemakers from sites such as the pulmonary veins. PVI remains central to radiofrequency ablation for AF. Successful ablation strategies aim to achieve electrical isolation of pulmonary veins by generating transmural lesions while minimizing collateral tissue damage. Consequently, lesion continuity, durability, and transmurality are critical determinants of isolation success (6). During RFA, electrical current rapidly heats superficial tissues, with heat gradually dissipating to deeper layers over time (17). Theoretically, the shorter energy delivery in HPSD may produce shallower lesions, raising concerns about incomplete transmurality (18) and potential AF recurrence (19). However, HPSD lesions achieve a minimum depth of 2.1 mm (20), exceeding the average left atrial wall thickness (1.5–2 mm), thus ensuring transmurality while reducing the risk of extra-cardiac tissue injury. Moreover, HPSD generates wider endocardial lesions than LPLD (21), enhancing continuity between adjacent lesions and promoting complete pulmonary vein encirclement. Yavin et al. (22) confirmed that HPSD significantly reduces chronic pulmonary vein reconnection rates compared with LPLD. Shorter energy delivery times may also improve catheter stability and contact force during respiration, mitigating transient suppression of electrical excitability by tissue edema to form ideal, durable lesions (23). Thus, in this study, HPSD significantly shortened procedure time and reduced AF recurrence. However, no statistical difference was observed between HPSD and LPLD in first-pass PVI.

However, HPSD raises safety considerations. The SHORT-AF trial observed a non-significant trend toward increased asymptomatic cerebral embolism (AE) with HPSD (24), potentially linked to char formation, thrombus, steam pops, or gas embolism at ablation sites (25). Zhang et al. (25) demonstrated in animal models that 50 W ablation generates more microembolic particles in cardiopulmonary bypass filters than 30 W ablation, likely due to endothelial damage exposing subendothelial collagen and tissue factor, triggering platelet activation and thrombosis. The same study found that drag ablation-with higher lesion overlap and tissue overheating produces more particles than point-by-point ablation, indicating a close association between radiofrequency application duration and AE formation. The clinical significance of asymptomatic cerebral embolism (ACE) remains unclear: von Bary et al. (26) reported no differences in neurocognitive function between patients with or without ACE post-ablation.

Long-term pulmonary vein reconnection is directly associated with atrial fibrillation recurrence and the need for repeat ablation and serves as a core endpoint for evaluating ablation durability. Yavin et al. conducted a retrospective study on patients who underwent repeat ablation, and the results showed that the chronic pulmonary vein reconnection rate in the HPSD group was significantly lower than that in the LPLD group (16.6% vs. 52.2%, *P* = 0.03) (22). This result is highly consistent with the conclusion of our meta-analysis that the atrial fibrillation recurrence rate was reduced in the HPSD group (*P* = 0.0004). Our study supports that HPSD may reduce the burden of repeat ablation. For example, the POWER-AF trial showed that none of the patients in the HPSD group (*n* = 48) required repeat ablation within 6 months, while three patients (6.25%) in the LPLD group (*n* = 48) did (14). In addition, a study by Barkagan et al. (23) found that the 2-year repeat ablation rate in the HPSD group was 12.3%, which was significantly lower than 21.7% in the LPLD group (*P* = 0.04), further verifying the long-term durability advantage of HPSD.

La Fazia et al. (27) conducted a prospective study that further validates the clinical value of radiofrequency ablation power settings, comparing the efficacy of three power strategies—25 W (LPLD), 40 W (medium power), and 50 W (HPSD)—for left atrial posterior wall ablation. It found that the transmural isolation rate in the HPSD group (92.5%) was significantly higher than that in the LPLD group (76.8%, *P* < 0.05), while the esophageal injury rate (4.2%) showed no statistical difference from the LPLD group (3.8%, *P* = 0.89). This result is consistent with the conclusion of our study that HPSD achieves a balance between efficacy and safety.

Pulsed-field ablation (PFA), as a rapidly developing non-thermal ablation technique in recent years, has become a research hotspot in atrial fibrillation ablation due to its core advantage of tissue-selective injury. Both PFA and HPSD radiofrequency ablation demonstrate high efficacy and safety in PVI for paroxysmal atrial fibrillation. Both techniques are comparable in terms of survival without arrhythmia. PFA offers shorter procedure times, providing added value in clinical settings. However, it involves longer fluoroscopy exposure and higher radiation doses. There is no significant difference in overall complication rates (28).

Although most of the included primary studies were of small sample size, their conclusions were generally consistent, and no publication bias was detected by Egger's test. This study performed TSA for each outcome. The curves for four outcomes—total procedure time, PVI time, radiofrequency application time, and AF recurrence—all exceeded the TSA boundary, confirming the efficacy of HPSD. However, the safety outcomes of HPSD did not reach the required sample size, necessitating more randomized controlled trial data.

## Conclusions

Compared with LPLD ablation, HPSD ablation significantly shortens the total procedure time, PVI time, and radiofrequency ablation time. HPSD ablation did not increase the risk of complications compared with LPLD ablation. However, this analysis may be underpowered to detect rare adverse events, necessitating additional large-scale RCTs to validate the safety profile of HPSD.

### Limitations

On the one hand, the relatively small sample sizes of the included studies may not accurately reflect the efficacy and safety of the different ablation strategies in a wider patient population, with the possibility of overestimating or underestimating the risk of events. On the other hand, the definitions of HPSD and LPLD, power parameters, catheter types, and ablation protocols varied between studies, which may lead to biased results and affect the accurate assessment of both ablation strategies. In addition, the heterogeneity of methods used to assess AF recurrence and the lack of subgroup analyses of different AF phenotypes (e.g., paroxysmal vs. persistent AF) limit insight into differences in ablation outcomes in different patient populations.

## Data Availability

The original contributions presented in the study are included in the article/Supplementary Material; further inquiries can be directed to the corresponding authors.
