# Supplementary material for: High-power short-duration vs. conventional catheter ablation for atrial fibrillation: a meta-analysis and trial sequential analysis of randomized controlled trials
Source: Front Cardiovasc Med. 2025 Dec 15;12:1703573. doi: 10.3389/fcvm.2025.1703573 (PMC12745485; doi:10.3389/fcvm.2025.1703573)
Supplement: Supplementary file 1 [file Datasheet1.pdf]

Table S1. Search strategy and results in Ovid MEDLINE database.

#	Searches	Results
**#1**	(("Atrial Fibrillation"[Mesh] OR ("atrial fibrillation"[All Fields] OR "AF"[All Fields]))	**111553**
**#2**	("Catheter Ablation"[Mesh] OR ("radiofrequency catheter ablation"[All Fields] OR "pulmonary vein isolation"[All Fields]))	**59031**
**#3**	("Catheter Ablation"[Mesh] OR ("radiofrequency catheter ablation"[All Fields] OR "pulmonary vein isolation"[All Fields]))	**534219**
**#4**	AND (("High - Power Short - Duration Ablation"[Mesh] OR ("HPSD"[All Fields] OR "High Power Short Duration"[All Fields]))	**3220842**
**#5**	AND ("Low - Power Long - Duration Ablation"[Mesh] OR ("LPLD"[All Fields] OR "Low Power Long Duration"[All Fields]))	
**#6**	**#1 AND #2 AND #3 NOT #4**	**1003**

Study or Subgroup	HPSD Mean	HPSD SD	HPSD Total	LPLD Mean	LPLD SD	LPLD Total	Weight	Mean Difference IV, Random, 95% CI	Year
**1.8.1 50-90W**									
Shin et al. 2020	122.15	29.6	100	161.9	37.9	50	14.0%	-39.75 [-51.75, -27.75]	2020
Francke et al. 2021	80.3	22.5	100	109.1	27.4	20	13.6%	-28.80 [-41.59, -16.01]	2021
POWER PLUS 2023	70	15.1	90	76.1	17.6	90	16.8%	-6.10 [-10.89, -1.31]	2023
SHORT-AF 2023	244.3	86.6	29	285	101.8	31	3.6%	-40.70 [-88.42, 7.02]	2023
POWER FAST III 2025	182	83.7	144	196	70.37	150	11.4%	-14.00 [-31.71, 3.71]	2025
**Subtotal (95% CI)**			**463**			**341**	**59.3%**	**-23.55 [-40.56, -6.54]**	
Heterogeneity: $Tau^2 = 289.74$; $Chi^2 = 34.22$, df = 4 (P < 0.00001); $I^2 = 88\%$									
Test for overall effect: Z = 2.71 (P = 0.007)									
**1.8.2 40-50W**									
PILOT-AF LSI4 2020	163.2	27.9	20	176.2	69.2	20	6.2%	-13.00 [-45.70, 19.70]	2020
PILOT-AF LSI5 2020	167.7	36	20	182.3	42.8	20	8.6%	-14.60 [-39.11, 9.91]	2020
POWER-AF 2021	89	29	48	107.7	26	48	14.4%	-18.70 [-29.72, -7.68]	2021
Hi-Lo HEAT 2023	133.3	37.6	44	150.9	45.8	44	11.5%	-17.60 [-35.11, -0.09]	2023
**Subtotal (95% CI)**			**132**			**132**	**40.7%**	**-17.58 [-26.01, -9.16]**	
Heterogeneity: $Tau^2 = 0.00$; $Chi^2 = 0.17$, df = 3 (P = 0.98); $I^2 = 0\%$									
Test for overall effect: Z = 4.09 (P < 0.0001)									
**Total (95% CI)**			**595**			**473**	**100.0%**	**-20.33 [-30.46, -10.21]**	
Heterogeneity: $Tau^2 = 153.28$; $Chi^2 = 35.33$, df = 8 (P < 0.0001); $I^2 = 77\%$									
Test for overall effect: Z = 3.94 (P < 0.0001)									
Test for subgroup differences: $Chi^2 = 0.38$, df = 1 (P = 0.54), $I^2 = 0\%$									

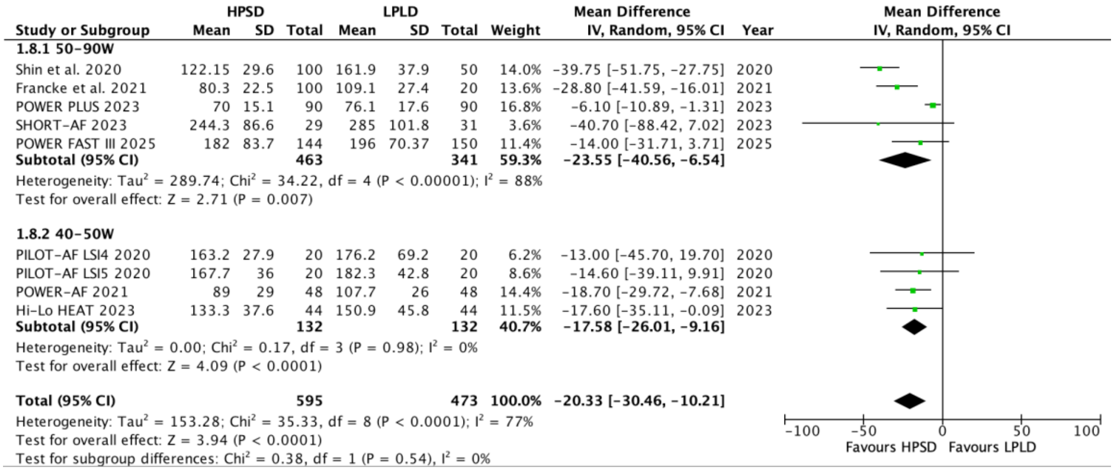

Figure S1. Subgroup analysis of total procedure time

Study or Subgroup	HPSD Events	HPSD Total	LPLD Events	LPLD Total	Weight	Risk Ratio M-H, Fixed, 95% CI	Year
**1.8.1 50-90W**							
Shin et al. 2020	10	98	5	48	10.5%	0.98 [0.35, 2.71]	2020
Francke et al. 2021	6	100	5	20	13.1%	0.24 [0.08, 0.71]	2021
SHORT-AF 2023	3	27	11	29	16.7%	0.29 [0.09, 0.94]	2023
**Subtotal (95% CI)**		**225**		**97**	**40.3%**	**0.46 [0.25, 0.83]**	
Total events	19		21				
Heterogeneity: $Chi^2 = 4.07$, df = 2 (P = 0.13); $I^2 = 51\%$							
Test for overall effect: Z = 2.55 (P = 0.01)							
**1.8.2 40-50W**							
PILOT-AF LSI4 2020	4	20	11	20	17.3%	0.36 [0.14, 0.95]	2020
PILOT-AF LSI5 2020	5	20	8	20	12.6%	0.63 [0.25, 1.58]	2020
POWER-AF 2021	5	48	4	48	6.3%	1.25 [0.36, 4.37]	2021
Hi-Lo HEAT 2023	7	44	15	44	23.6%	0.47 [0.21, 1.03]	2023
**Subtotal (95% CI)**		**132**		**132**	**59.7%**	**0.55 [0.35, 0.88]**	
Total events	21		38				
Heterogeneity: $Chi^2 = 2.60$, df = 3 (P = 0.46); $I^2 = 0\%$							
Test for overall effect: Z = 2.50 (P = 0.01)							
**Total (95% CI)**		**357**		**229**	**100.0%**	**0.51 [0.36, 0.74]**	
Total events	40		59				
Heterogeneity: $Chi^2 = 6.99$, df = 6 (P = 0.32); $I^2 = 14\%$							
Test for overall effect: Z = 3.54 (P = 0.0004)							
Test for subgroup differences: $Chi^2 = 0.25$, df = 1 (P = 0.62), $I^2 = 0\%$							

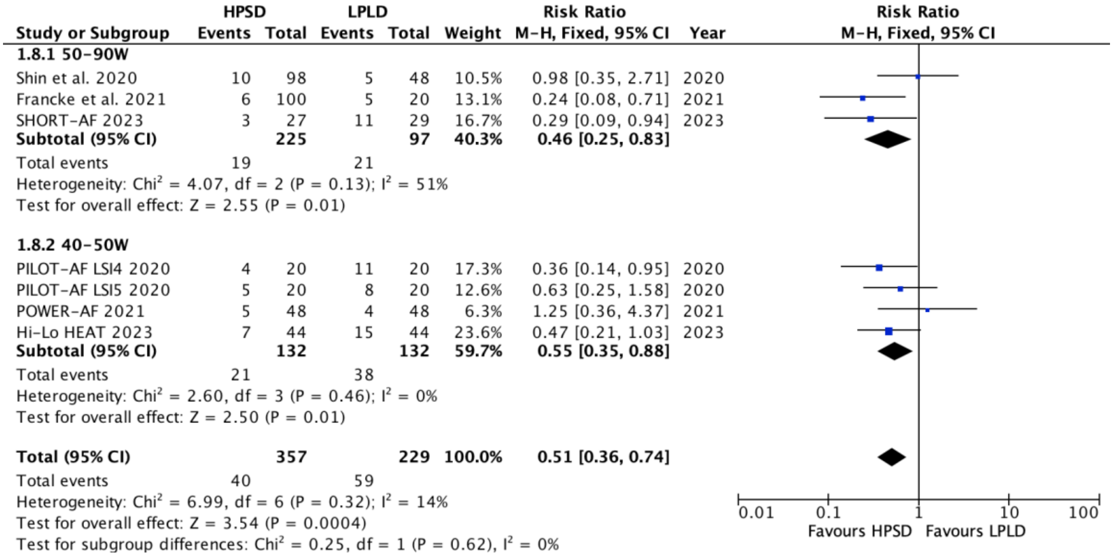

Figure S2. Subgroup analysis of AF recurrence

Study or Subgroup	HPSD Events	HPSD Total	LPLD Events	LPLD Total	Weight	Odds Ratio M-H, Fixed, 95% CI	Year
Shin et al. 2020	15	98	9	48	18.6%	0.78 [0.32, 1.95]	2020
Hi-Lo HEAT 2023	19	44	16	44	16.6%	1.33 [0.56, 3.13]	2023
SHORT-AF 2023	3	29	11	31	0.0%	0.21 [0.05, 0.85]	2023
POWER PLUS 2023	15	90	13	90	19.7%	1.18 [0.53, 2.66]	2023
POWER FAST III 2025	43	144	36	150	45.1%	1.35 [0.80, 2.26]	2025
**Total (95% CI)**		**376**		**332**	**100.0%**	**1.21 [0.84, 1.73]**	
Total events	92		74				
Heterogeneity: $Chi^2 = 1.10$, df = 3 (P = 0.78); $I^2 = 0\%$							
Test for overall effect: Z = 1.03 (P = 0.30)							

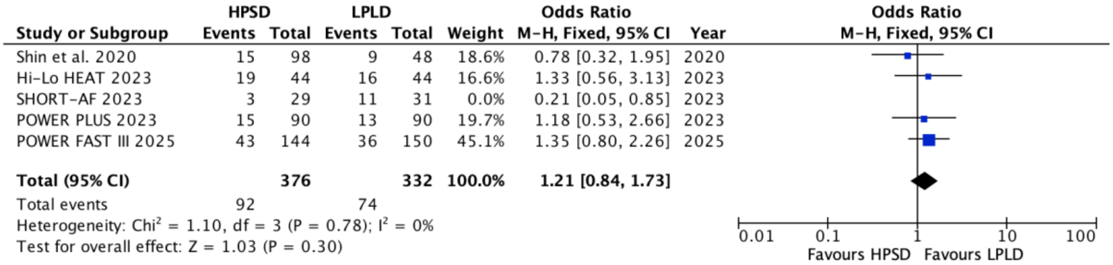

Figure S3. Sensitivity analysis of all atrial arrhythmias recurrence

Study or Subgroup	HPSD Events	HPSD Total	LPLD Events	LPLD Total	Weight	Risk Ratio M-H, Fixed, 95% CI	Year
Francke et al. 2021	84	100	10	20	0.0%	1.68 [1.07, 2.63]	2021
POWER-AF 2021	46	48	41	48	14.6%	1.12 [0.98, 1.28]	2021
POWER PLUS 2023	76	90	81	90	28.8%	0.94 [0.84, 1.05]	2023
Hi-Lo HEAT 2023	28	44	31	44	11.0%	0.90 [0.67, 1.21]	2023
POWER FAST III 2025	118	144	131	150	45.6%	0.94 [0.85, 1.03]	2025
**Total (95% CI)**		**326**		**332**	**100.0%**	**0.96 [0.90, 1.03]**	
Total events	268		284				

Heterogeneity: $Chi^2 = 5.94$, df = 3 (P = 0.11); $I^2 = 50\%$
Test for overall effect: Z = 1.17 (P = 0.24)

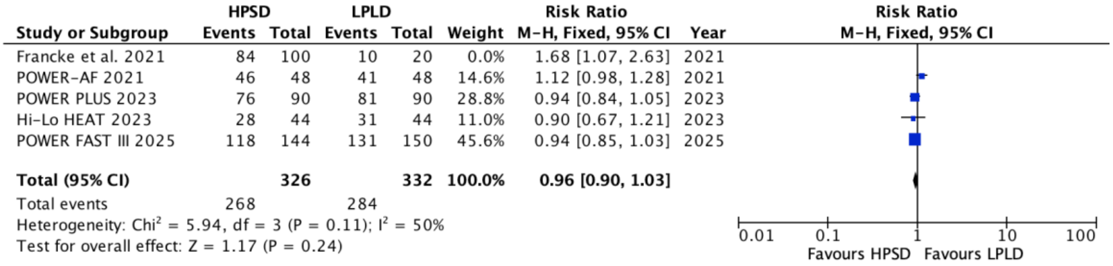

Figure S4. Sensitivity analysis of first pass RPV isolation

Study or Subgroup	HPSD Events	HPSD Total	LPLD Events	LPLD Total	Weight	Risk Ratio M-H, Fixed, 95% CI	Year
**1.8.1 50-90W**							
Shin et al. 2020	0	50	0	50		Not estimable	2020
Francke et al. 2021	13	97	2	20	29.6%	1.34 [0.33, 5.48]	2021
POWER PLUS 2023	1	90	1	90	8.9%	1.00 [0.06, 15.74]	2023
SHORT-AF 2023	0	29	0	31		Not estimable	2023
POWER FAST III 2025	5	139	4	146	34.8%	1.31 [0.36, 4.79]	2025
**Subtotal (95% CI)**		**405**		**337**	**73.3%**	**1.29 [0.52, 3.16]**	
Total events	19		7				
Heterogeneity: $Chi^2 = 0.04$, df = 2 (P = 0.98); $I^2 = 0\%$							
Test for overall effect: Z = 0.55 (P = 0.58)							
**1.8.2 40-50W**							
PILOT-AF LSI4 2020	0	20	0	20		Not estimable	2020
PILOT-AF LSI5 2020	0	20	0	20		Not estimable	2020
POWER-AF 2021	1	48	1	48	8.9%	1.00 [0.06, 15.53]	2021
Hi-Lo HEAT 2023	2	44	2	44	17.8%	1.00 [0.15, 6.79]	2023
**Subtotal (95% CI)**		**132**		**132**	**26.7%**	**1.00 [0.21, 4.81]**	
Total events	3		3				
Heterogeneity: $Chi^2 = 0.00$, df = 1 (P = 1.00); $I^2 = 0\%$							
Test for overall effect: Z = 0.00 (P = 1.00)							
**Total (95% CI)**		**537**		**469**	**100.0%**	**1.21 [0.55, 2.64]**	
Total events	22		10				

Heterogeneity: $Chi^2 = 0.11$, df = 4 (P = 1.00); $I^2 = 0\%$
Test for overall effect: Z = 0.48 (P = 0.63)
Test for subgroup differences: $Chi^2 = 0.07$, df = 1 (P = 0.79), $I^2 = 0\%$

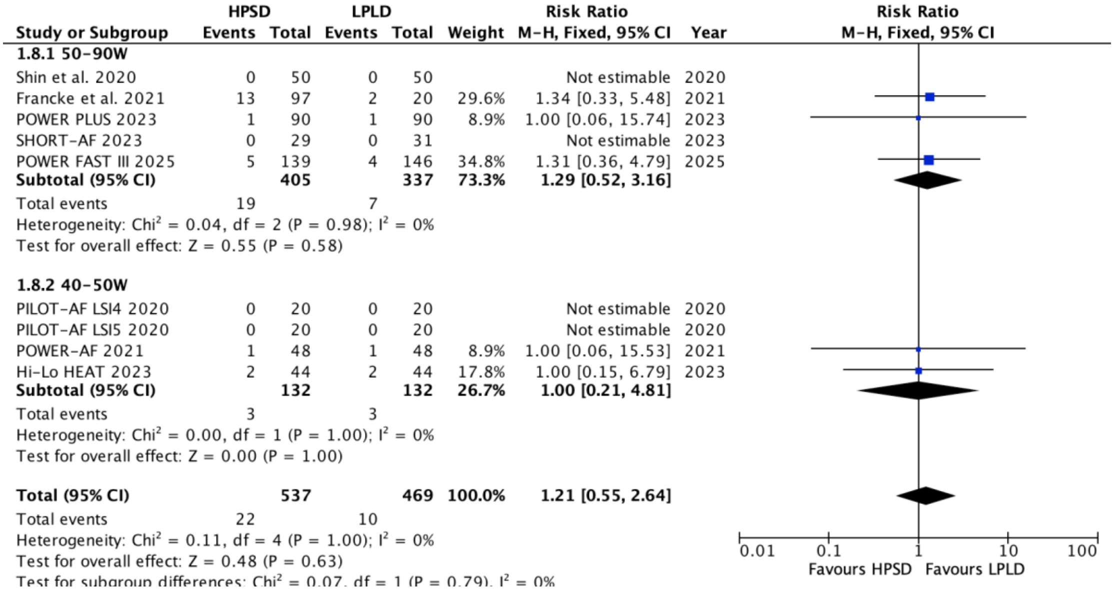

Figure S5. Subgroup analysis of esophageal lesions

Study or Subgroup	HPSD Events	HPSD Total	LPLD Events	LPLD Total	Weight	Risk Ratio M-H, Fixed, 95% CI	Year
**1.8.1 50-90W**							
Shin et al. 2020	1	100	0	50	3.5%	1.51 [0.06, 36.53]	2020
Francke et al. 2021	13	97	2	20	17.7%	1.34 [0.33, 5.48]	2021
POWER PLUS 2023	2	90	2	90	10.6%	1.00 [0.14, 6.95]	2023
SHORT-AF 2023	1	29	1	31	5.1%	1.07 [0.07, 16.31]	2023
POWER FAST III 2025	11	144	8	150	41.7%	1.43 [0.59, 3.46]	2025
**Subtotal (95% CI)**		**460**		**341**	**78.7%**	**1.33 [0.69, 2.58]**	
Total events	28		13				
Heterogeneity: $Chi^2 = 0.14$, df = 4 (P = 1.00); $I^2 = 0\%$							
Test for overall effect: Z = 0.85 (P = 0.39)							
**1.8.2 40-50W**							
PILOT-AF LSI4 2020	1	20	0	20	2.7%	3.00 [0.13, 69.52]	2020
PILOT-AF LSI5 2020	1	20	0	20	2.7%	3.00 [0.13, 69.52]	2020
POWER-AF 2021	1	48	1	48	5.3%	1.00 [0.06, 15.53]	2021
Hi-Lo HEAT 2023	2	44	2	44	10.6%	1.00 [0.15, 6.79]	2023
**Subtotal (95% CI)**		**132**		**132**	**21.3%**	**1.50 [0.43, 5.19]**	
Total events	5		3				
Heterogeneity: $Chi^2 = 0.63$, df = 3 (P = 0.89); $I^2 = 0\%$							
Test for overall effect: Z = 0.64 (P = 0.52)							
**Total (95% CI)**		**592**		**473**	**100.0%**	**1.37 [0.76, 2.45]**	
Total events	33		16				

Heterogeneity: $Chi^2 = 0.78$, df = 8 (P = 1.00); $I^2 = 0\%$
Test for overall effect: Z = 1.06 (P = 0.29)
Test for subgroup differences: $Chi^2 = 0.03$, df = 1 (P = 0.87), $I^2 = 0\%$

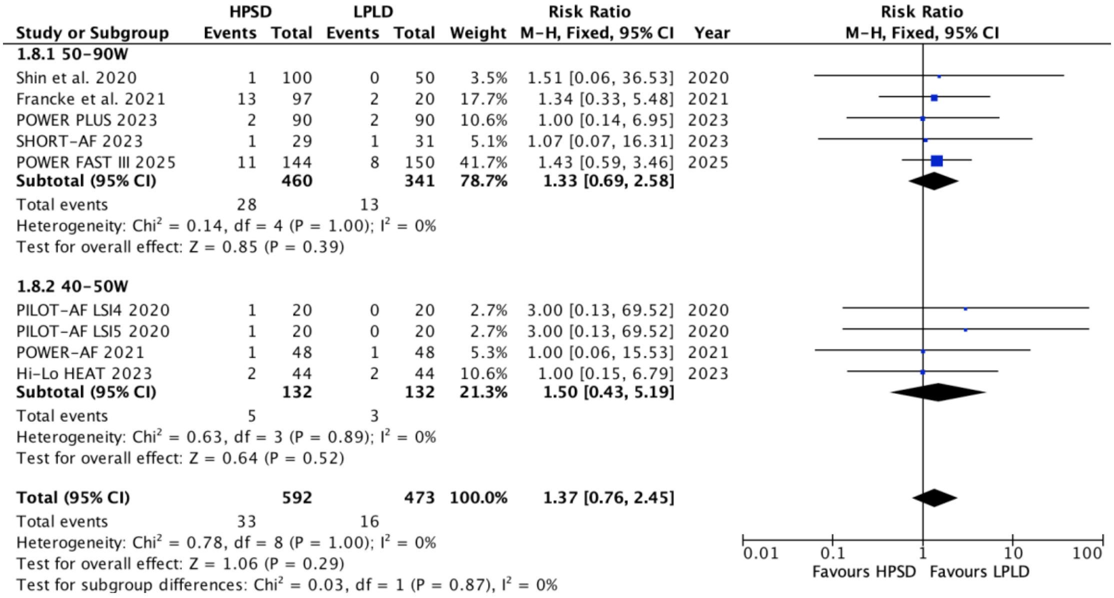

Figure S6. Subgroup analysis of any complications

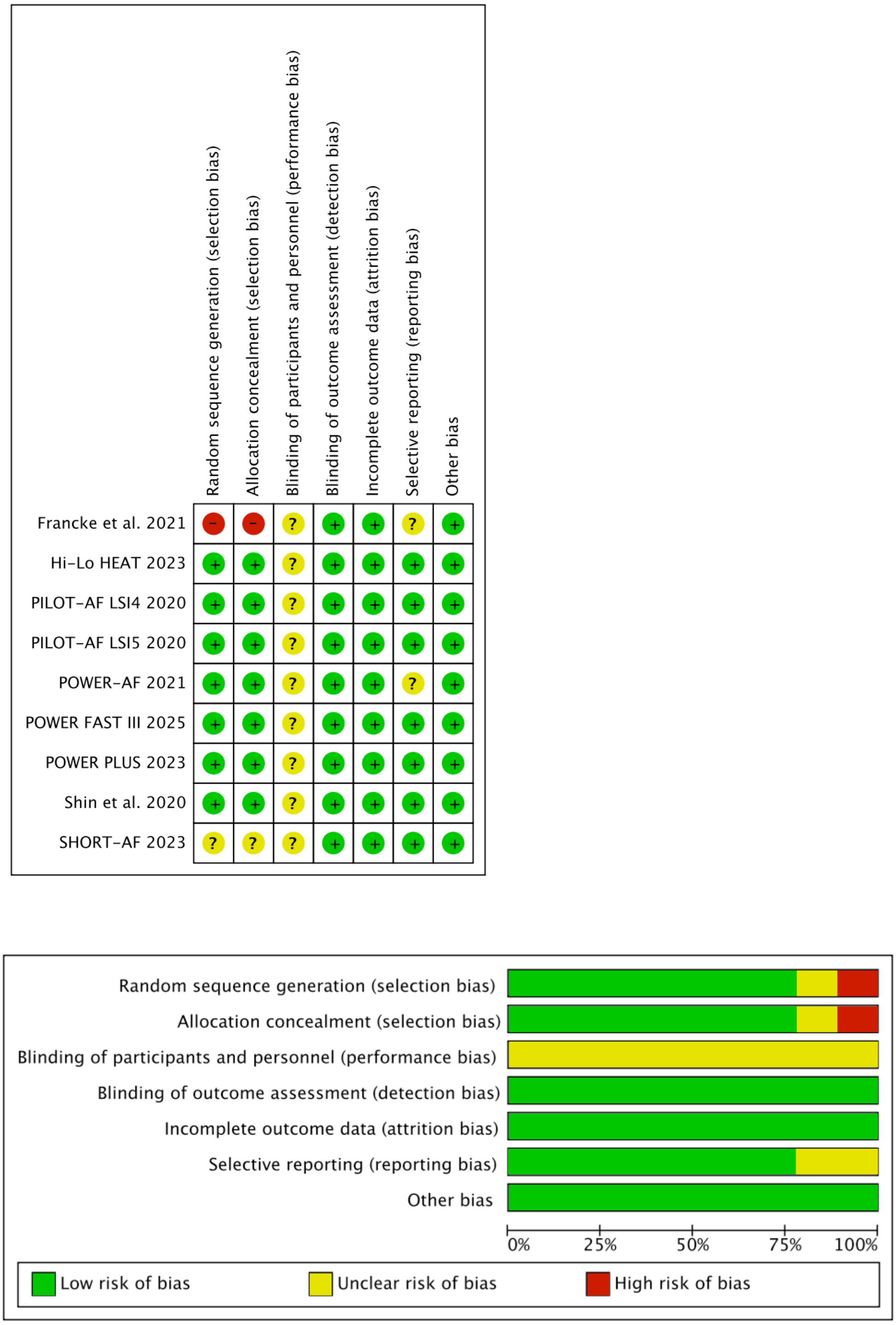

Figure S7. Risk of Bias Assessment

A **B**

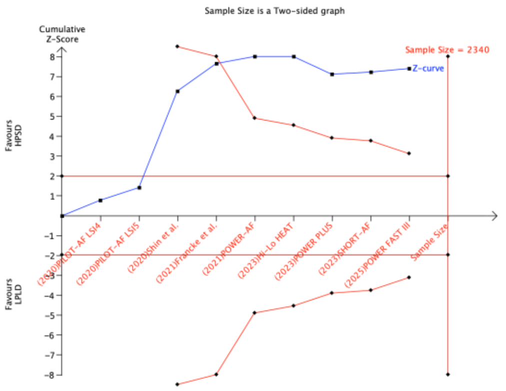

Sample Size is a Two-sided graph
Cumulative Z-Score
Favours HPSD
Favours LPLD
Sample Size = 2340
Z-curve
(2020)PILOT-AF LSI4
(2020)PILOT-AF LSI5
(2020)Shin et al.
(2021)Francke et al.
(2021)POWER-AF
(2023)Hi-Lo HEAT
(2023)POWER PLUS
(2023)SHORT-AF
(2025)POWER FAST III
Sample Size

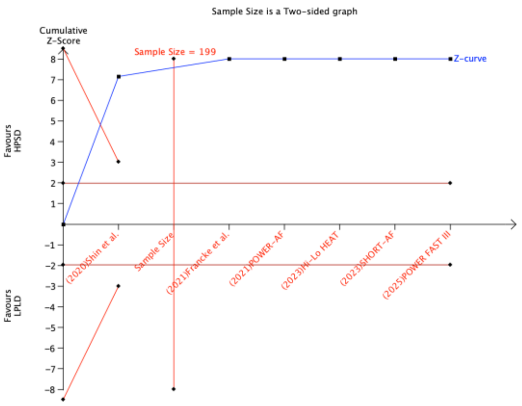

Sample Size is a Two-sided graph
Cumulative Z-Score
Favours HPSD
Favours LPLD
Sample Size = 199
Z-curve
(2020)Shin et al.
Sample Size
(2021)Francke et al.
(2021)POWER-AF
(2023)Hi-Lo HEAT
(2023)SHORT-AF
(2025)POWER FAST III

C **D**

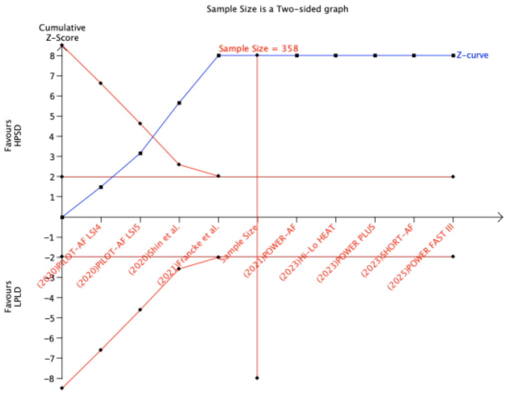

Sample Size is a Two-sided graph
Cumulative Z-Score
Favours HPSD
Favours LPLD
Sample Size = 358
Z-curve
(2020)PILOT-AF LSI4
(2020)PILOT-AF LSI5
(2020)Shin et al.
(2021)Francke et al.
Sample Size
(2021)POWER-AF
(2023)Hi-Lo HEAT
(2023)POWER PLUS
(2023)SHORT-AF
(2025)POWER FAST III

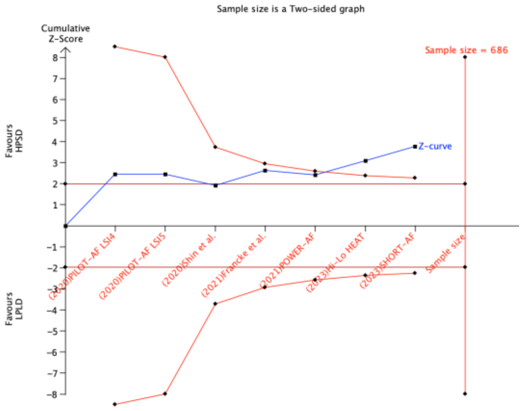

Sample size is a Two-sided graph
Cumulative Z-Score
Favours HPSD
Favours LPLD
Sample size = 686
Z-curve
(2020)PILOT-AF LSI4
(2020)PILOT-AF LSI5
(2020)Shin et al.
(2021)Francke et al.
(2021)POWER-AF
(2023)Hi-Lo HEAT
(2023)SHORT-AF
Sample size

E **F**

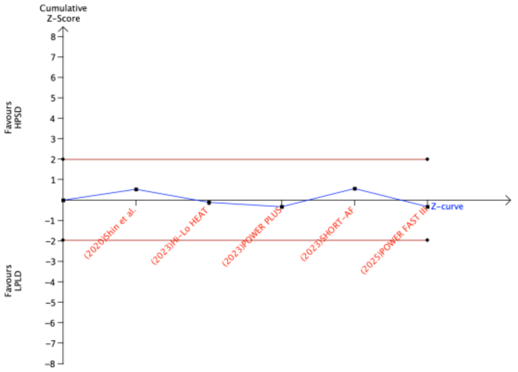

Cumulative Z-Score
Favours HPSD
Favours LPLD
Z-curve
(2020)Shin et al.
(2023)Hi-Lo HEAT
(2023)POWER PLUS
(2023)SHORT-AF
(2025)POWER FAST III

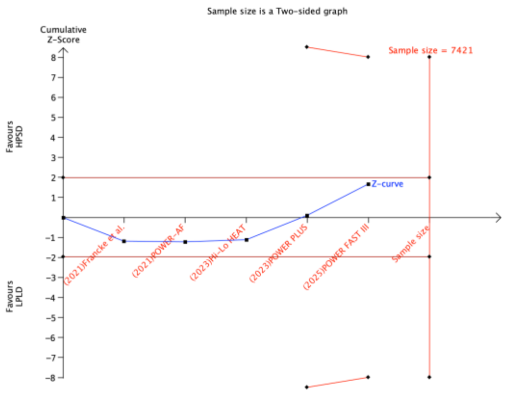

Sample size is a Two-sided graph
Cumulative Z-Score
Favours HPSD
Favours LPLD
Sample size = 7421
Z-curve
(2021)Francke et al.
(2021)POWER-AF
(2023)Hi-Lo HEAT
(2023)POWER PLUS
(2025)POWER FAST III
Sample size

G **H**

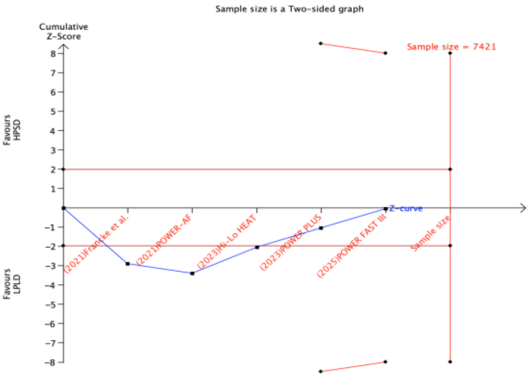

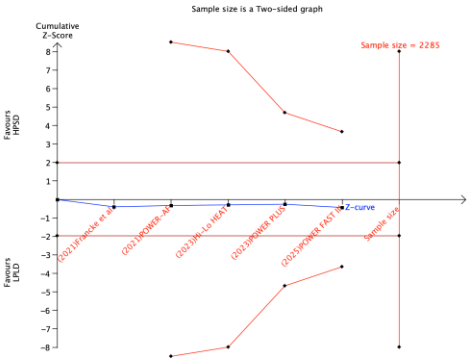

I

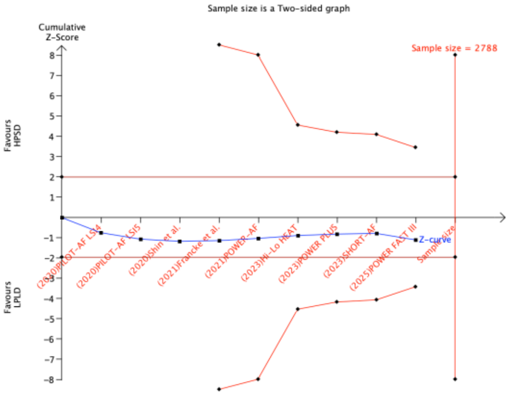

Figure S8. Trial sequential analysis of each outcome.

A: Total procedure time, B: PVI time, C: Radiofrequency application time, D: AF recurrence, E: All atrial arrhythmias recurrence, F: First-pass LPV isolation, G: First-pass RPV isolation, H: Esophageal lesions, I: Any complications.

,

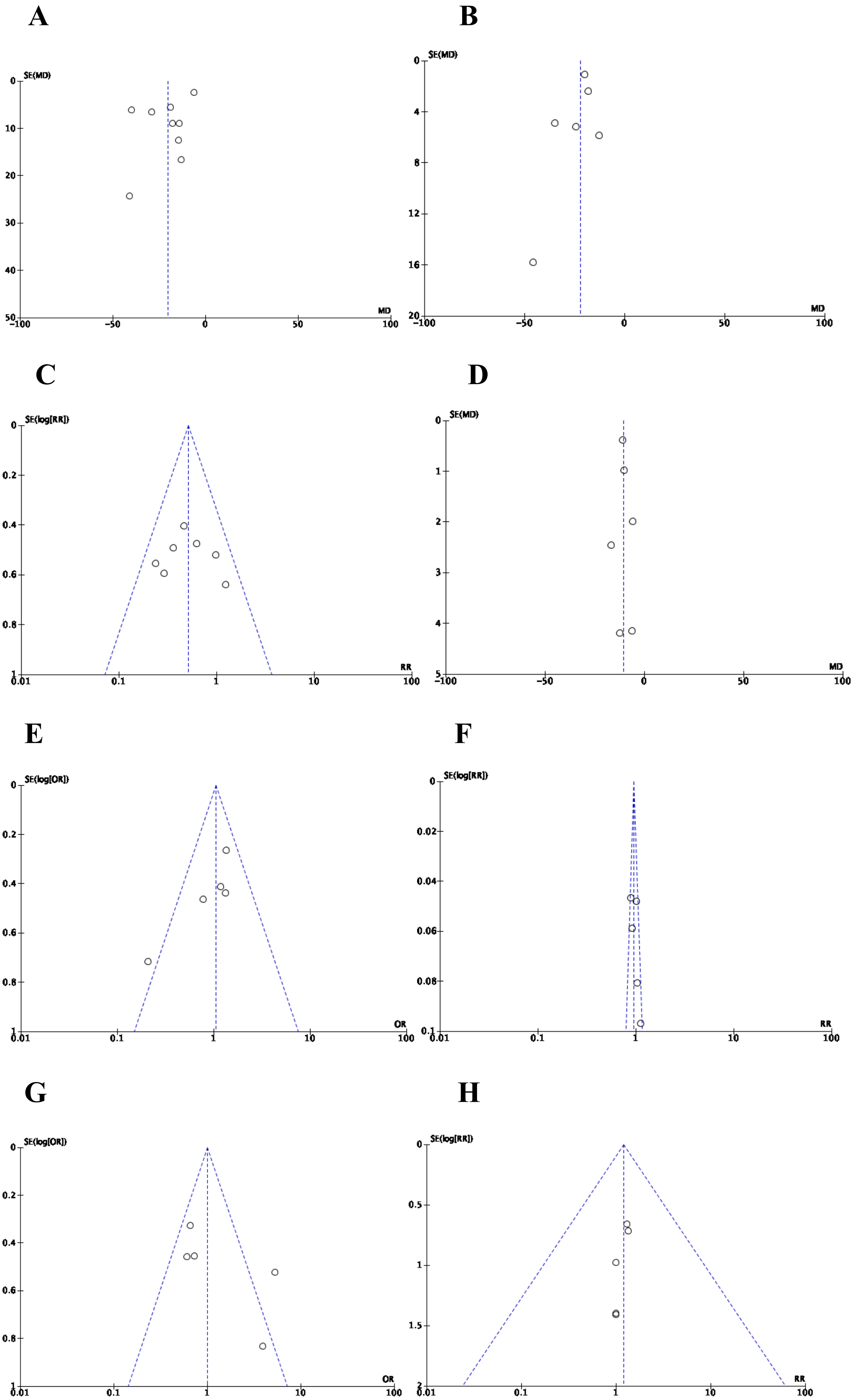

A
SE(MD)
MD
B
SE(MD)
MD
C
SE(log[RR])
RR
D
SE(MD)
MD
E
SE(log[OR])
OR
F
SE(log[RR])
RR
G
SE(log[OR])
OR
H
SE(log[RR])
RR

I

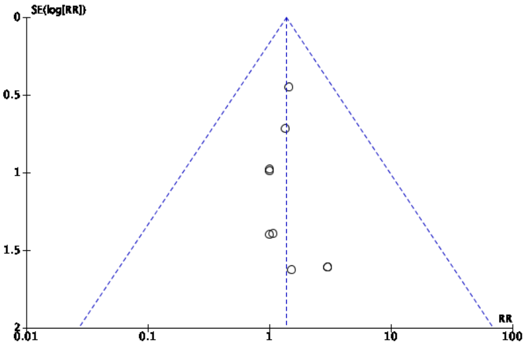

Figure S9. Funnel plot of each outcome.

A: Total procedure time, B: PVI time, C: Radiofrequency application time, D: AF recurrence, E: All atrial arrhythmias recurrence, F: First-pass LPV isolation, G: First-pass RPV isolation, H: Esophageal lesions, I: Any complications.

Table S2. The result of and certainty of evidence assessment

Certainty assessment						No of patients		Effect	Certainty
**No of studies**	**Study design**	**Risk of bias**	**Inconsistency**	**Imprecision**	**Other**	**LPSD**	**LPLD**	**MD/RR**	
**Total procedure time**									
**9**	Randomized trials	Not serious	moderate	moderate	None	595	473	-20.33(-30.46 to -10.21)	moderate
**Pulmonary vein isolation time**									
**6**	Randomized trials	Not serious	Not serious	moderate	None	412	339	-22.01(-27.10 to -16.95)	High
**Radiofrequency time**									
**6**	Randomized trials	Not serious	Not serious	Not serious	None	322	242	-10.38 (-12.47 to -8.29)	High
**AF recurrence**									
**7**	Randomized trials	Not serious	moderate	Not serious	None	40/357	59/229	0.51 (0.36-0.74)	High
All atrial arrhythmias recurrence									
**5**	Randomized trials	Not serious	moderate	Not serious	None	95/405	85/363	1.06 (0.75-1.49)	High
Esophageal lesions									
**9**	Randomized trials	Not serious	Not serious	moderate	None	22/537	10/469	1.21 (0.55, 2.64)	High
Any complications									
**9**	Randomized trials	Not serious	Not serious	moderate	None	33/592	16/473	1.37 (0.76, 2.45)	High
First pass LPV isolation									
**5**	Randomized trials	Not serious	Not serious	Not serious	None	373/426	318/352	0.96 (0.91, 1.01)	High
First pass RPV isolation									
**5**	Randomized trials	Not serious	moderate	Not serious	None	22/537	10/469	1.01 (0.69, 1.48)	High

Table S3 Meta-Regression of total procedure time.

Covariate	Regression Coefficient	Standard Error (SE)	95% Confidence Interval	Z-value	P-value
AF type	1.28	0.45	[0.39, 2.17]	2.84	0.004
Power of LPLD	-0.04	0.07	[-0.18, 0.10]	-0.57	0.568
Power of HPSD	0.04	0.02	[0.01, 0.07]	2.35	0.019
CHA2DS2-VASc Score	0.42	0.18	[0.07, 0.77]	2.11	0.035
Age (years)	-0.02	0.04	[-0.10, 0.06]	-0.50	0.616
LVEF (%)	0.05	0.03	[0.00, 0.10]	1.96	0.050
Follow-up Duration	0.15	0.32	[-0.48, 0.78]	0.47	0.638

Table S4. Meta-Regression of PVI time.

Covariate	Regression Coefficient	Standard Error (SE)	95% Confidence Interval	Z-value	P-value
AF type	-14.80	5.62	[-25.82, -3.78]	-2.63	0.008
Power of LPLD	0.19	0.33	[-0.46, 0.84]	0.58	0.561
Power of HPSD	-0.36	0.16	[-0.67, -0.05]	-2.25	0.024
CHA2DS2-VASc Score	-3.20	1.35	[-5.85, -0.55]	-2.37	0.018
Age (years)	0.12	0.39	[-0.65, 0.89]	0.31	0.756
LVEF (%)	0.29	0.26	[-0.22, 0.80]	1.12	0.262
Follow-up Duration	-1.85	2.95	[-7.64, 3.94]	-0.63	0.528

Table S5. Meta-Regression of radiofrequency application time.

Covariate	Regression Coefficient	Standard Error (SE)	95% Confidence Interval	Z-value	P-value
AF type	-0.49	0.20	[-0.88, -0.10]	-2.45	0.014
Power of LPLD	0.01	0.03	[-0.05, 0.07]	0.33	0.740
Power of HPSD	-0.02	0.01	[-0.04, -0.00]	-2.01	0.044
CHA2DS2-VASc Score	-0.25	0.11	[-0.47, -0.03]	-2.27	0.023
Age (years)	0.01	0.02	[-0.03, 0.05]	0.50	0.617
LVEF (%)	-0.01	0.01	[-0.03, 0.01]	-1.00	0.317
Follow-up Duration	-0.20	0.14	[-0.47, 0.07]	-1.43	0.152

Table S6. The results of trial sequential analysis of each outcome.

outcomes	RIS	Cumulative Z-curve position	RIS
Total procedure time	2340	Beyond the conventional and TSA boundary, not reach RIS	2340
PVI time	199	Beyond the conventional and TSA boundary and RIS	199
Radiofrequency application time	686	Beyond the conventional and TSA boundary, not reach RIS	358
AF recurrence	358	Beyond the TSA boundary and RIS	686
All atrial arrhythmias recurrence		Not beyond the conventional and TSA boundary	
First-pass LPV isolation	7421	Not beyond the conventional and TSA boundary	7421
First-pass RPV isolation	7421	Not beyond the conventional and TSA boundary	7421
Esophageal lesions	2285	Not beyond the conventional and TSA boundary	2285
Any complications	2788	Not beyond the conventional and TSA boundary	2788

RIS, required information size.

Table S7. The Bgger's test of each outcome.

	Z	P
**Total procedure time**	-0.282	0.778
**Pulmonary vein isolation time**	−0.192	0.848
Radiofrequency time	1.243	0.214
AF recurrence	0.26	0.76
All atrial arrhythmias recurrence	0.85	0.39
Esophageal lesions	0.72	0.47
Any complications	0.91	0.36
First pass LPV isolation	−1.409	0.159
First pass RPV isolation	1.687	0.091
